# High Glucose-Induced PC12 Cell Death by Increasing Glutamate Production and Decreasing Methyl Group Metabolism

**DOI:** 10.1155/2016/4125731

**Published:** 2016-06-19

**Authors:** Minjiang Chen, Hong Zheng, Tingting Wei, Dan Wang, Huanhuan Xia, Liangcai Zhao, Jiansong Ji, Hongchang Gao

**Affiliations:** ^1^School of Pharmaceutical Sciences, Wenzhou Medical University, Wenzhou 325035, China; ^2^Lishui Central Hospital, The Fifth Affiliated Hospital, Wenzhou Medical University, Lishui 323000, China

## Abstract

*Objective*. High glucose- (HG-) induced neuronal cell death is responsible for the development of diabetic neuropathy. However, the effect of HG on metabolism in neuronal cells is still unclear.* Materials and Methods*. The neural-crest derived PC12 cells were cultured for 72 h in the HG (75 mM) or control (25 mM) groups. We used NMR-based metabolomics to examine both intracellular and extracellular metabolic changes in HG-treated PC12 cells.* Results*. We found that the reduction in intracellular lactate may be due to excreting more lactate into the extracellular medium under HG condition. HG also induced the changes of other energy-related metabolites, such as an increased succinate and creatine phosphate. Our results also reveal that the synthesis of glutamate from the branched-chain amino acids (isoleucine and valine) may be enhanced under HG. Increased levels of intracellular alanine, phenylalanine, myoinositol, and choline were observed in HG-treated PC12 cells. In addition, HG-induced decreases in intracellular dimethylamine, dimethylglycine, and 3-methylhistidine may indicate a downregulation of methyl group metabolism.* Conclusions*. Our metabolomic results suggest that HG-induced neuronal cell death may be attributed to a series of metabolic changes, involving energy metabolism, amino acids metabolism, osmoregulation and membrane metabolism, and methyl group metabolism.

## 1. Introduction

Diabetes mellitus is a group of metabolic disorder diseases affecting an increasing number of the population in the world. According to survey results from Shaw et al. [1], approximately 6.4% of the global adult population suffered from diabetes mellitus in 2010, and this number will increase to 7.7% by 2030. The development of macro- and microvascular complications is the main cause of the morbidity and mortality of diabetes [2]. Diabetic neuropathy is the most common microvascular complication of diabetes and more than half of diabetics will develop neuropathy, which affects sensory, motor, and autonomic nerves and results in nontraumatic amputations and autonomic failure [3]. For symptom management, clinical evidence supports that diabetic neuropathy can be treated with an array of medications mainly including anticonvulsants and antidepressants [4].

Hyperglycemia, as a common diabetes symptom, plays a great important role in the development of diabetic neuropathy [5]. Hyperglycemia induces oxidative stress in neurons, which in turn results in neuronal cell apoptosis and dysfunction [6]. An increased flux of the polyol pathway in the peripheral nerve tissues caused by hyperglycemia has been associated with the development of neuropathy [7]. Thornalley [8] reported that every component in diabetic nerves can be glycated and thereby resulted in neuropathy. Kamiya et al. [9] found a reduced protein kinase C (PKC) activity in Schwann cells cultured under high glucose. PKC has been shown to play a key role in nerve function and impact the development of diabetic neuropathy [10]. The lack of neurotrophins [11] and proinflammatory processes [12] are also related to diabetic neuropathy. Moreover, hyperglycemia was also found to affect the nerve conduction velocity [13] and alter the pattern of neurotransmitter release in the cerebral cortex [14]. Although the pathological causes of diabetic neuropathy are increasingly discovered, the potential metabolic mechanism of high glucose-induced toxicity on neuronal cells is still far from being fully understood.

The PC12 cell, as a mature cell line from a rat pheochromocytoma, has been commonly used as a cellular model for studying the mechanisms underlying neuronal cell death and neurotransmitter secretion [15] as well as glucose neurotoxicity [16, 17]. Yürekli et al. have used 1-methyl-4-phenylpyridinium-ion-induced PC12 cells as a cellular model of Parkinson disease to study the effect of Zonisamide on oxidative stress and caspase activity [18]. PC12 cells were also used to explore the protective effect of telmisartan on high glucose-induced neurotoxicity [19]. In the present study, a ^1^H NMR-based metabolomics method was applied to examine both intracellular and extracellular metabolic changes in PC12 cells under high glucose in order to explore underlying metabolic mechanisms of high glucose-induced neuronal cell death.

## 2. Materials and Methods

### 2.1. Materials and Reagents

PC12 cells were obtained from the Chemical Biology Research Center, Wenzhou Medical University. Dulbecco's modified Eagle's medium (DMEM), fetal bovine serum (FBS), and 0.25% trypsin-EDTA (1x) were purchased from GIBCO BRL (Eggenstein, Germany). Penicillin-streptomycin solution containing 10.000 units/mL penicillin and streptomycin and phosphate buffer saline (PBS) were purchased from HyClone. Methanol and chloroform were purchased from the Sinopharm Chemical Reagent Co., Ltd. (analytical grade). In addition, ultrapure water was prepared using the Millipore MilliQ purification system.

### 2.2. Cell Number and Viability Assays

**Figure 1:**
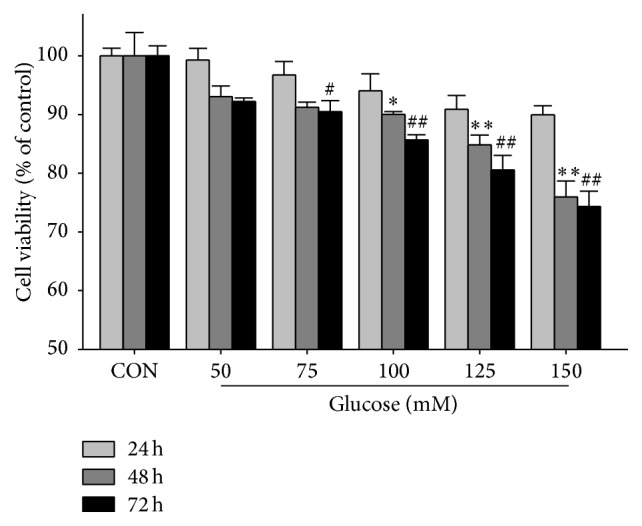
Effects of glucose concentration and culture time on PC12 cell viability. Data are presented as mean ± SEM (*n* = 3). The difference between the control (CON) group and the treatment group with different concentrations of glucose after 24, 48, and 72 h. Significant level: ^*∗*^ *p* < 0.05, ^*∗∗*^ *p* < 0.01, ^#^ *p* < 0.05, ^##^ *p* < 0.01.

**Figure 2:**
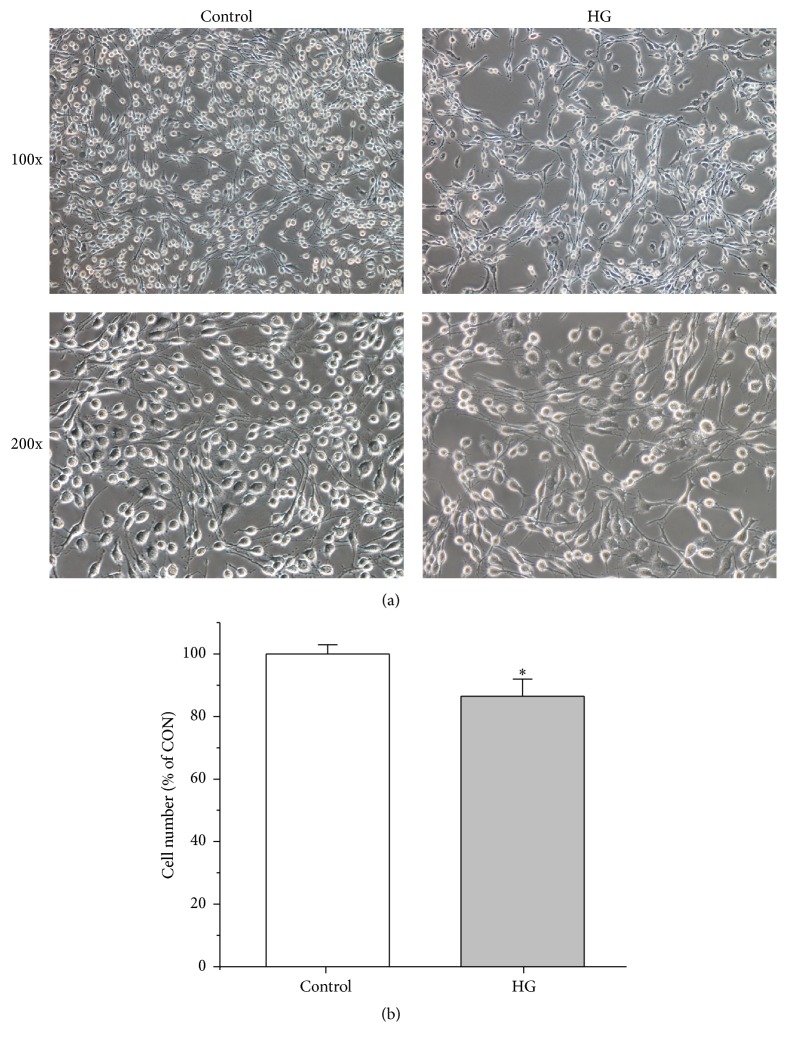
The number of PC12 cells in the control (25 mM) and high glucose (HG, 75 mM) conditions: (a) representative micrographs of PC12 cells taken by a light microscope (Nikon Eclipse Ti); (b) the change in PC12 cell number counted using Image-Pro Plus 6.0 (Media Cybernetics, Bethesda, MA). Significant level: ^*∗*^ *p* < 0.05.

The cell viability was assessed with 3-(4,5-dimethylthiazol-2-yl)2,5-diphenyl-tetrazolium bromide (MTT) assay for the selection of high glucose culture condition. In brief, PC12 cells were seeded at a density of 5,000 per well in 96-well microplates. After overnight incubation, the culture media were replaced with fresh DMEM media containing different concentrations of glucose (25, 50, 75, 100, 125, and 150 mM) and PC12 cells were cultured for 24, 48, and 72 h, respectively. Subsequently, each culture well was added with 20 *μ*L MTT (0.5 mg/mL) and placed at 37°C for 4 h. Then, the medium was removed carefully and 150 *μ*L DMSO was added in each well to dissolve the formazan product. Finally, formazan absorbance was assessed at a wavelength of 570 nm using a microplate reader (Multiskan Mk3; Thermo Labsystems, Helsinki, Finland). Each experiment was repeated three times.  Figure 1 illustrates the change of cell viability with increases of glucose concentration and culture time. We found that the cell viability was significantly decreased when PC12 cells were cultured with 75 mM of glucose relative to 25 mM of glucose for 72 h (Figure 1). Furthermore, the number of PC12 cells in these two conditions for 72 h was also determined using a light microscope (Nikon eclipse Ti). In this study, we randomly selected six different horizons at 200 times for counting the number of PC12 cells by Image-Pro Plus 6.0 (Media Cybernetics, Bethesda, MA).  Figure 2 shows that the number of PC12 cells was significantly decreased in 75 mM of glucose for 72 h as compared with 25 mM of glucose. Therefore, 75 mM of glucose and 72 h were selected as the high glucose (HG) condition and culture time for further studies, respectively.

PC12 cells were routinely thawed and cultured in T-75 culture flasks with DMEM media containing 25 mM glucose, 10% FBS, 1% penicillin, and 1% streptomycin in a humidified atmosphere containing 5% CO_2_ at 37°C. When confluent, these cells were trypsinized and seeded in 12 T-175 culture flasks (5 × 10^5^ cells per flask). After 24 h seeding, PC12 cells were washed three times by PBS, and the culture media were replaced by fresh DMEM media containing 25 mM glucose as the control group (*n* = 6) and 75 mM as the HG group (*n* = 6). To reduce the hyperosmolar effect of glucose, 50 mmol/L mannitol was supplemented in the control group. After 72 h of culture, these cells were trypsinized, centrifuged at 300 ×g for 5 min at 4°C, and harvested for metabolomic analysis. Meanwhile, the corresponding culture media were also collected for NMR analysis.

### 2.3. Intracellular Metabolites Extraction

The harvested cells were washed three times with cold PBS and then methanol-chloroform-water extraction was used as previously described [20]. Briefly, cell pellets were resuspended in 500 *μ*L of ice-cold 2 : 1 (v/v) methanol/chloroform solution and then transferred into a 1.5 mL Eppendorf tube. After vortexing, the tubes were incubated on a mixer for 10 min at 4°C. Then, 250 *μ*L of ice-cold 1 : 1 (v/v) chloroform/H_2_O was added and mixed using a vortex mixer. The mixture was ultrasonicated on ice for 10 min and centrifuged at 13,000 ×g for 20 min at 4°C. The supernatant was collected, lyophilized, and stored at −80°C until analysis.

### 2.4. Extracellular Metabolites Extraction

Metabolites were extracted both from the blank medium (DMEM + 10% FBS) and from the conditioned culture medium using a modified Bligh-Dyer extraction method [21]. Briefly, 1 mL culture medium was transferred from each culture flask into individual 15 mL centrifuge tubes. Then, 3 mL of ice-cold methanol/chloroform solution (v/v 2 : 1) was added and followed by 1 mL of ice-cold chloroform. The mixture was centrifuged at 11,000 ×g for 20 min at 4°C for the separation between the upper (methanol/water) and lower (organic) phase. The upper phase fraction was freeze-dried and stored at −80°C for further analysis.

### 2.5. ^1^H NMR Measurement

**Figure 3:**
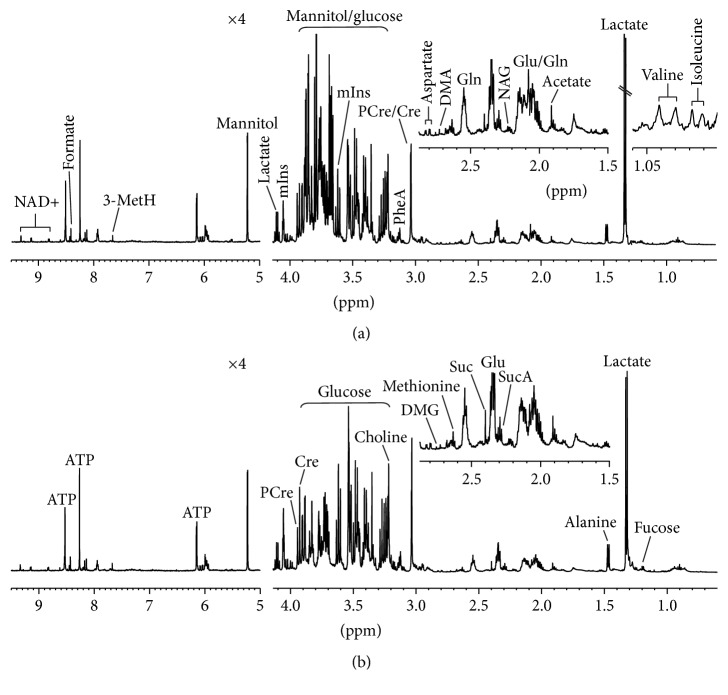
Representative 600 MHz 1D ^1^H NMR spectra obtained from intracellular extracts in the control (a) and HG (b) groups. The abbreviation of metabolites: NAG: N-acetylglutamate; SucA: succinylacetone; Glu: glutamate; Suc: succinate; Gln: glutamine; DMA: dimethylamine; DMG: dimethylglycine; PCre: creatine phosphate; Cre: creatine; PheA: phenylalanine; mIns: myoinositol; 3-MetH: 3-methylhistidine.

The lyophilized intracellular and extracellular extracts were reconstituted with 600 *μ*L D_2_O and centrifuged at 12,000 ×g for 10 min at 4°C. Then, 550 *μ*L aliquots of the supernatant were pipetted into 5 mm NMR tubes for ^1^H NMR experiments. All ^1^H NMR spectra were recorded at 25°C using a Bruker AVANCE III 600 spectrometer operating at 600.13 MHz equipped with a triple resonance probe. A one-dimensional ZGPR pulse sequence was applied to achieve satisfactory water suppression in aqueous extracts. Moreover, the other main acquisition parameters included data points, 32 K; relaxation delay, 8 sec; spectral width, 12,000 Hz; number of scans, 512; acquisition time, 2.66 sec per scan; exponential line-broadening function, 0.3 Hz.  Figure 3 and Figure S1, in Supplementary Material available online at http://dx.doi.org/10.1155/2016/4125731, illustrate the typical ^1^H NMR spectra obtained from intracellular and extracellular extracts, respectively.

### 2.6. Spectral Preprocessing and Multivariate Data Analysis

All spectra were phase- and baseline-corrected and referenced to the methyl peak of lactate (CH_3_) at 1.33 ppm. Then, the spectra were integrated to binning data with a size of 0.01 ppm from 0.4 to 10.0 ppm excluding the residual water region from 4.66 to 5.02 ppm by using the Bruker Topspin 2.1 software (v2.1 pl4, Bruker Biospin, Germany). Finally, prior to multivariate data analysis, the binned data of each NMR spectrum were normalized to the total sum of the spectral intensity.

Partial least squares-discriminate analysis (PLS-DA) was performed to identify the separation between the control and HG groups using the SIMCA 13.0 software (Umetrics, Umeå, Sweden). Leave-one-out cross validation (LOOCV) and permutation tests (200 cycles) were conducted to assess the performance of PLS-DA. The explained variance (*R* ^2^) and the predictive ability (*Q* ^2^) of the model were calculated and these two parameters close to 1.0 mean an excellent model. In addition, the absolute value of the correlation coefficient, |*r*|, in PLS-DA was used to identify variables mainly contributing to the separation between two groups.

### 2.7. Statistical Analysis

Analysis of variance (ANOVA) was applied to evaluate the difference between the control and HG groups using SPSS software (version 16.0). The difference was considered statistically significant when *p* < 0.05.

## 3. Results

### 3.1. Effects of Glucose Concentration and Culture Time on PC12 Cell Viability

The change of PC12 cell viability with increases in glucose concentration and culture time was shown in Figure 1. PC12 cell viability was significantly inhibited when cultured for 72 h with >75 mM of glucose or for 48 h with >100 mM of glucose as compared with controls (25 mM of glucose). Results from electron micrographs also demonstrated that the number of PC12 cells was significantly reduced after culturing for 72 h with 75 mM of glucose relative to 25 mM of glucose (Figure 2).

### 3.2. Metabolomic Analysis of Intracellular Metabolites

**Figure 4:**
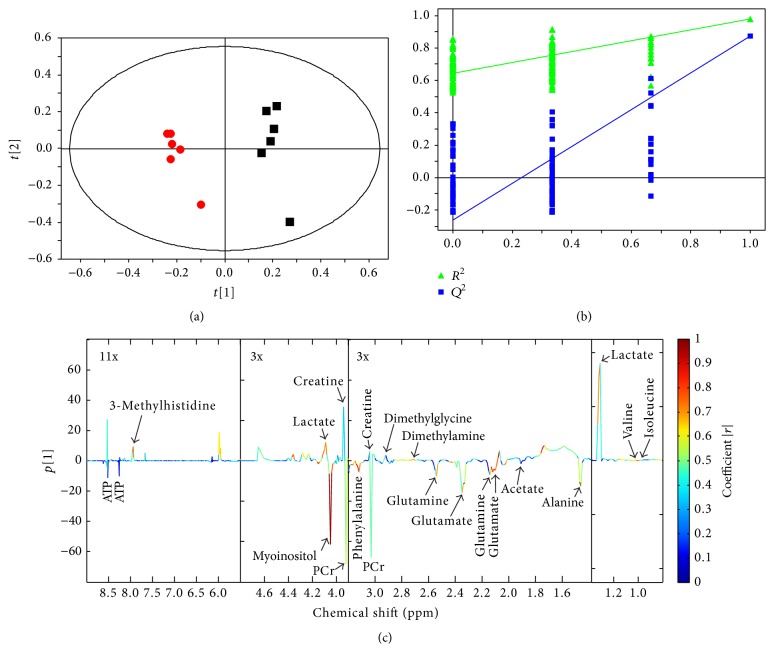
PLS-DA results obtained from NMR-based intracellular metabolome in the control (■) and HG (●) groups: (a) score plot; (b) validation plot by permutation tests (200 cycles); (c) loading plot colored according to the absolute value of the correlation coefficient.

Figure 3 illustrates typical ^1^H NMR spectra detected from intracellular extracts in the control and HG groups, and a total of 26 metabolites were identified, involving energy metabolism, amino acid metabolism, osmoregulation, and membrane metabolism as well as methyl group metabolism. Then, multivariate data analysis (PLS-DA) was employed to analyze the metabolic difference between the control and HG groups. Results based on PLS-DA show that the HG group was clearly separated from the control group (Figure 4(a)) and the model was validated by permutation tests (Figure 4(b)).  Figure 4(c) illustrates the corresponding loading plot colored according to the significance of classification from the correlation matrix, and coefficient values were listed in Table 1. Moreover, Table 1 also shows the results of univariate data analysis (ANOVA) between the control and HG groups. It can be seen from Table 1 that HG-treated PC12 cells had higher concentrations of alanine (17.94 ± 0.42 versus 13.77 ± 0.42), glutamate (44.36 ± 0.58 versus 38.69 ± 0.72), succinate (3.03 ± 0.07 versus 2.60 ± 0.16), creatine phosphate (21.42 ± 0.43 versus 18.63 ± 0.47), phenylalanine (17.33 ± 0.36 versus 15.46 ± 0.33), choline (31.04 ± 0.48 versus 26.67 ± 0.68), and myoinositol (61.02 ± 1.22 versus 47.27 ± 1.26) but lower levels of isoleucine (0.91 ± 0.01 versus 1.06 ± 0.03), valine (1.42 ± 0.03 versus 1.65 ± 0.08), dimethylamine (0.72 ± 0.03 versus 0.90 ± 0.04), dimethylglycine (0.63 ± 0.03 versus 0.79 ± 0.04), aspartate (3.15 ± 0.12 versus 3.74 ± 0.17), lactate (19.87 ± 0.15 versus 22.83 ± 0.63), and 3-methylhistidine (0.88 ± 0.05 versus 1.07 ± 0.06) as compared with the control cells.

### 3.3. Metabolomic Analysis of Extracellular Metabolites

Figure S1 shows typical ^1^H NMR spectra obtained from extracellular extracts in the control and HG groups. To further examine the metabolic change in neurons under HG condition, PLS-DA was also performed on the basis of the ^1^H NMR spectra obtained from extracellular media between the control and HG groups, as shown in Figure S2. The clear separation between them suggests that metabolic perturbation also occurs outside the neuron. In the present study, relative changes of metabolite levels in the extracellular media cultured with control and HG cells compared with the blank media were calculated as listed in Table S1. According to multivariate and univariate analyses, we found that the HG treatment reduced isoleucine (−9.57 ± 0.08 versus −8.22 ± 0.11), methionine (−2.31 ± 0.05 versus −2.01 ± 0.04), lysine (−9.34 ± 0.28 versus −7.30 ± 0.21), myoinositol (−2.26 ± 0.16 versus −1.66 ± 0.10), threonine (−2.03 ± 0.05 versus −1.24 ± 0.08), tyrosine (−2.77 ± 0.03 versus −2.65 ± 0.03), phenylalanine (−1.90 ± 0.03 versus −1.44 ± 0.03), and tryptophan (−0.41 ± 0.05 versus −0.27 ± 0.03) more than the control in the extracellular media, while opposite results were obtained in valine (−6.96 ± 0.19 versus −9.07 ± 0.60), ethanol (−20.92 ± 3.39 versus −32.02 ± 1.96), creatine phosphate (−0.35 ± 0.07 versus −0.63 ± 0.02), creatine (−0.13 ± 0.04 versus −0.42 ± 0.02), and choline (−1.18 ± 0.07 versus −1.81 ± 0.05). In addition, relative to the blank media, acetate level (−0.12 ± 0.19 versus 0.44 ± 0.05) in the extracellular media was increased in the control group but decreased in the HG group. Table S1 shows that both of the control and HG groups had increased levels of alanine (2.57 ± 0.13 versus 7.35 ± 0.15) and lactate (31.54 ± 0.36 versus 25.84 ± 0.51) compared with the blank media. However, the increased amount of lactate in the HG group (31.54 ± 0.36 versus 25.84 ± 0.51) was significantly higher than that in the control group, while the HG group had a lower amount of increased alanine level (2.57 ± 0.13 versus 7.35 ± 0.15), as shown in Table S1.

## 4. Discussion

Hyperglycemia plays a critical role in the development of diabetic neuropathy, which may be attributed to HG-induced neuronal cell death [22]. In this study, we found that the viability and number of PC12 cells were significantly decreased in the HG group (75 mM of glucose) compared with the control group (25 mM of glucose). However, the potential metabolic mechanisms are still unknown. Therefore, NMR-based metabolomics was used to examine the metabolic changes inside and outside the HG-treated PC12 cells, and the results show that HG-induced neuronal cell death may be associated with the changes of energy metabolism, amino acids metabolism, osmoregulation, and membrane metabolism as well as methyl group metabolism.

### 4.1. Effect of HG on Energy Metabolism in PC12 Cells

Cellular energy metabolism is closely linked with its functional activity. In neuronal cells, lactate has been demonstrated as an energy source by Wyss et al. [23]. Moreover, Avogaro et al. [24] reported that lactate plays a key role in providing energy for the brain other than glucose during hypoglycemia. Results of the present study show that intracellular lactate level was significantly decreased in the HG group compared with the control group, indicating that the HG treatment may reduce lactate production in PC12 cells. In our previous study, a reduction in lactate level was observed in both of astrocytes and neurons in* db/db* mice compared with wild-type mice [25]. Our data also show that both groups had a significantly increased level of extracellular lactate, while a higher lactate was observed in the HG medium relative to the control medium. Therefore, under HG condition, PC12 cells may also excrete more lactate into the extracellular medium, which resulted in a further decrease in intracellular lactate level.

However, our data reveal that succinate, as a TCA cycle intermediate, was significantly increased in PC12 cells under HG condition relative to control condition. Moreover, we also found a higher creatine phosphate level in the HG-treated PC12 cells relative to the control cells. Creatine phosphate is a phosphorylated creatine that serves as a reserve pool of high-energy phosphates and is utilized for ATP formation in conditions of high-energy demand [26]. Therefore, this study suggests that energy metabolism in the HG-treated PC12 cells was mainly derived from glucose metabolism, but not lactate metabolism.

### 4.2. Effect of HG on Amino Acids Metabolism in PC12 Cells

The branched-chain amino acids (BCAAs) such as isoleucine and valine are important nitrogen sources for glutamate synthesis via transamination [27]. In the present study, intracellular isoleucine and valine levels were significantly decreased in the HG group and meanwhile glutamate concentration was significantly increased, indicating that the glutamate synthesis from the BCAAs may be enhanced under HG state. In addition, according to intracellular and extracellular changes in alanine level, we found that the amount of alanine released into the medium was lower under HG condition as compared with that under control condition. Waagepetersen et al. [28] reported that alanine is a possible nitrogen carrier between glutamatergic neurons and astrocytes and thereby provides nitrogen for glutamate/glutamine synthesis in astrocytes. Hence, a higher intracellular level of alanine may be responsible for an increase in glutamate in the HG-treated PC12 cells. In our previous study, we also found an increased level of glutamate in neurons of* db/db* mice as indicated by enhanced enrichments in the C2, C3, and C4 of glutamate after [3-^13^C]-lactate infusion [25]. Glutamate is the major excitatory neurotransmitter in the central nervous system (CNS) [29]. The glutamate-glutamine cycle between astrocytes and neurons can regulate synaptic glutamate homeostasis and thereby maintain normal CNS function [30]. Thus, HG-induced increase in glutamate is probably one of the reasons for an increased PC12 cell death under HG condition. However, the level of aspartate, as another excitatory neurotransmitter, was decreased in the HG-treated PC12 cells relative to the control cells in this study. This finding is in disagreement with our* in vivo* mice study, where a significant increase in aspartate was observed in the brain of* db/db* mice as compared with wild-type mice [25].

In addition, intracellular phenylalanine level was increased and the corresponding extracellular concentration was reduced under HG condition. A high phenylalanine level has been found to cause cell death in cultured neurons [31], which is related to the RhoA/Rho-associated kinase pathway [32]. Therefore, our data suggest that phenylalanine-induced PC12 cell death may also occur under HG condition.

### 4.3. Effect of HG on Osmoregulation and Membrane Metabolism in PC12 Cells

Osmoregulation plays a vital role in the maintenance of cell structure and function [33]. Myoinositol has been reported as an important osmolyte in astrocytes [34]. In the present study, we found that intracellular myoinositol level was increased in the HG cells relative to the control cells, which may be caused by an afflux of extracellular myoinositol. van der Graaf et al. [35] reported that the level of myoinositol was increased in the hippocampus of diabetic fatty rats compared to controls in order to avoid ion-induced perturbation of protein function. Moreover, myoinositol was also found to be increased in neurological disorders [36, 37]. Choline is essential for membrane phospholipid synthesis in all cells [38]. An increase in intracellular choline level in the HG-treated PC12 cells and a reduction of choline in the corresponding extracellular media were observed in our study, indicating the accumulation of choline for assuring the structural integrity of cell membranes. Therefore, HG-induced increases in myoinositol and choline may suggest a self-protection behavior of PC12 cells under HG stress.

### 4.4. Effect of HG on Methyl Group Metabolism in PC12 Cells

Disturbance of methyl group metabolism has been implicated in various pathological conditions [39]. For instance, Wainfan and Poirier [40] found that the intake of methyl-deficient diets resulted in hepatocarcinogenesis in rats by inducing DNA methylation and aberrant gene expression. Defective methyl group metabolism may be associated with the neurological damage induced by HIV infection [41]. In addition, methyl groups were also disrupted in both type 1 and type 2 diabetes [41, 42]. Therefore, maintenance of methyl group metabolism homeostasis plays an important role in human health. However, in our* in vitro* study, we found that HG resulted in reduced intracellular dimethylamine, dimethylglycine, and 3-methylhistidine levels as compared with the control group, suggesting that a downregulation of methyl group metabolism may be induced by HG stress. Formate can be produced from many ways. In mitochondria, it is mainly derived from serine and glycine as well as two intermediates of choline catabolism, sarcosine and dimethylglycine [42]. In the present study, therefore, a significant decrease in formate level in the HG-treated PC12 cells may be attributed to a reduction in dimethylglycine under HG condition. Therefore, we speculate that a decrease in methyl group metabolism may also be responsible for HG-induced PC12 cell death.

Several limitations need to be considered as follows: (1) PC12 is a mature cell line from a rat pheochromocytoma, but it could be of great interest to use a primary neuronal cell line to study the mechanisms underlying HG-induced cell death; (2) these findings were based on an* in vitro* study, so it needs to be further verified by* in vivo* studies; (3) metabolites were limited to be detected by a single analytical technique, so an integrated analytical platform such as LC-MS and GC-MS is recommended to achieve a more detailed metabolic analysis; (4) the present results were only based on metabolomic analysis but combining with genomic or proteomic analysis will advance deep understanding of the mechanisms underlying HG-induced neuronal cell death.

## 5. Conclusions

In the present study, we expectedly found that HG induced PC12 cells death. Thus, NMR-based metabolomics was applied to explore the metabolic mechanisms underlying this phenomenon. A disturbance of energy metabolism and a reduction in methyl group metabolism were found in the HG-treated PC12 cells. HG treatment may enhance glutamate synthesis from BCAAs, resulting in the increase in glutamate level in PC12 cells. Moreover, our results suggest that phenylalanine-induced PC12 cells death may also occur under HG stress. However, increases in myoinositol and choline may indicate a self-protection behavior of PC12 cells under HG stress. The* in vitro* results proposed in the present study may advance understanding of the pathogenesis of diabetic neuropathy. However, these findings should be further verified in* in vivo* studies.

## Supplementary Material

In the Supplementary Material, typical ^1^H NMR spectra in extracellular extracts of the control and high glucose (HG) groups were illustrated in Figure S1. A clear separation was observed between the control and HG groups by multivariate analysis (PLS-DA) based on the extracellular metabolite profiles and the contributed metabolites were identified from its loading plot (Figure S2). In addition, Table S1 lists the changes in extracellular metabolite levels between the control and HG groups.

## Figures and Tables

**Table 1:** Comparison of intracellular metabolites levels between the control and HG groups.

Number	Metabolites	|*r*|^a^	CON^b^	HG^c^
1	Isoleucine	0.56	1.06 ± 0.03	0.91 ± 0.01^*∗∗∗*^
2	Valine	0.87	1.65 ± 0.08	1.42 ± 0.03^*∗*^
3	Fucose	0.46	4.34 ± 0.26	4.70 ± 0.10
4	Alanine	0.95	13.77 ± 0.42	17.94 ± 0.42^*∗∗∗*^
5	Acetate	0.20	4.21 ± 0.19	4.43 ± 0.37
6	N-Acetylglutamate	0.21	4.52 ± 0.28	4.57 ± 0.20
7	Succinylacetone	0.56	2.19 ± 0.06	2.19 ± 0.05
8	Glutamate	0.92	38.69 ± 0.72	44.36 ± 0.58^*∗∗∗*^
9	Succinate	0.62	2.60 ± 0.16	3.03 ± 0.07^*∗*^
10	Glutamine	0.64	25.52 ± 0.68	26.47 ± 0.54
11	Methionine	0.38	4.35 ± 0.12	4.25 ± 0.08
12	Dimethylamine	0.71	0.90 ± 0.04	0.72 ± 0.03^*∗∗*^
13	Dimethylglycine	0.53	0.79 ± 0.04	0.63 ± 0.03^*∗*^
14	Aspartate	0.70	3.74 ± 0.17	3.15 ± 0.12^*∗*^
15	Creatine phosphate	0.54	18.63 ± 0.47	21.42 ± 0.43^*∗∗*^
16	Creatine	0.37	14.89 ± 0.87	12.74 ± 0.37
17	Phenylalanine	0.88	15.46 ± 0.33	17.33 ± 0.36^*∗∗*^
18	Choline	0.88	26.67 ± 0.68	31.04 ± 0.48^*∗∗∗*^
19	Myoinositol	0.98	47.27 ± 1.26	61.02 ± 1.22^*∗∗∗*^
20	Lactate	0.72	22.83 ± 0.63	19.87 ± 0.15^*∗∗*^
21	3-Methylhistidine	0.55	1.07 ± 0.06	0.88 ± 0.05^*∗*^
22	Formate	0.32	0.54 ± 0.02	0.44 ± 0.02^*∗∗*^

^a^The absolute value of correlation coefficient obtained from PLS-DA; ^b^control group (25 mM glucose); ^c^high glucose group (75 mM glucose). Significant level: ^*∗*^ *p* < 0.05, ^*∗∗*^ *p* < 0.01, ^*∗∗∗*^ *p* < 0.001.

## References

[1] Shaw J. E., Sicree R. A., Zimmet P. Z. (2010). Global estimates of the prevalence of diabetes for 2010 and 2030. *Diabetes Research and Clinical Practice*.

[2] Hirakawa Y., Arima H., Zoungas S. (2014). Impact of visit-to-visit glycemic variability on the risks of macrovascular and microvascular events and all-cause mortality in type 2 diabetes: the ADVANCE trial. *Diabetes Care*.

[3] Brock C. (2014). Associations between sensorimotor, autonomic and central neuropathies in diabetes mellitus. *Journal of Diabetes & Metabolism*.

[4] Callaghan B. C., Cheng H. T., Stables C. L., Smith A. L., Feldman E. L. (2012). Diabetic neuropathy: clinical manifestations and current treatments. *The Lancet Neurology*.

[5] Katagi M., Terashima T., Okano J. (2014). Hyperglycemia induces abnormal gene expression in hematopoietic stem cells and their progeny in diabetic neuropathy. *FEBS Letters*.

[6] Kumar P., Raman T., Swain M. M., Mishra R., Pal A. (2016). Hyperglycemia-induced oxidative-nitrosative stress induces inflammation and neurodegeneration via augmented tuberous sclerosis complex-2 (TSC-2) activation in neuronal cells. *Molecular Neurobiology*.

[7] Obrosova I. G., Ilnytska O., Lyzogubov V. V. (2007). High-fat diet-induced neuropathy of pre-diabetes and obesity: effects of ‘healthy’ diet and aldose reductase inhibition. *Diabetes*.

[8] Thornalley P. J. (2002). Glycation in diabetic neuropathy: characteristics, consequences, causes, and therapeutic options. *International Review of Neurobiology*.

[9] Kamiya H., Nakamura J., Hamada Y. (2003). Polyol pathway and protein kinase C activity of rat Schwannoma cells. *Diabetes/Metabolism Research and Reviews*.

[10] Geraldes P., King G. L. (2010). Activation of protein kinase C isoforms and its impact on diabetic complications. *Circulation Research*.

[11] Nazıroğlu M., Dikici D. M., Dursun Ş. (2012). Role of oxidative stress and Ca^2+^ signaling on molecular pathways of neuropathic pain in diabetes: focus on TRP channels. *Neurochemical Research*.

[12] Yerra V. G., Negi G., Sharma S. S., Kumar A. (2013). Potential therapeutic effects of the simultaneous targeting of the Nrf2 and NF-*κ*B pathways in diabetic neuropathy. *Redox Biology*.

[13] Yadav N., Shete A., Yadav P., Yadav N., Khan S. T. (2015). Study of nerve conduction velocity in type II diabetes mellitus. *National Journal of Integrated Research in Medicine*.

[14] Lacoste B., Comin C. H., Ben-Zvi A. (2014). Sensory-related neural activity regulates the structure of vascular networks in the cerebral cortex. *Neuron*.

[15] Li B.-R., Hsieh Y.-J., Chen Y.-X., Chung Y.-T., Pan C.-Y., Chen Y.-T. (2013). An ultrasensitive nanowire-transistor biosensor for detecting dopamine release from living pc12 cells under hypoxic stimulation. *Journal of the American Chemical Society*.

[16] Liu M.-H., Yuan C., He J. (2015). Resveratrol protects PC12 cells from high glucose-induced neurotoxicity via PI3K/Akt/FoxO3a pathway. *Cellular and Molecular Neurobiology*.

[17] Najafi R., Sharifi A. M., Hosseini A. (2015). Protective effects of alpha lipoic acid on high glucose-induced neurotoxicity in PC12 cells. *Metabolic Brain Disease*.

[18] Yürekli V. A., Gürler S., Nazıroğlu M., Uğuz A. C., Koyuncuoğlu H. R. (2013). Zonisamide attenuates MPP(+)-induced oxidative toxicity through modulation of Ca^2+^ signaling and caspase-3 activity in neuronal PC12 cells. *Cellular and Molecular Neurobiology*.

[19] Eslami H., Sharifi A. M., Rahimi H., Rahati M. (2014). Protective effect of telmisartan against oxidative damage induced by high glucose in neuronal PC12 cell. *Neuroscience Letters*.

[20] Gottschalk M., Ivanova G., Collins D. M., Eustace A., O'Connor R., Brougham D. F. (2008). Metabolomic studies of human lung carcinoma cell lines using *in vitro*  
^1^H NMR of whole cells and cellular extracts. *NMR in Biomedicine*.

[21] Miccheli A., Ricciolini R., Piccolella E., Delfini M., Conti F. (1991). Modulation of human lymphoblastoid B cell line by phorbol ester and sphingosine. A 31P-NMR study. *Biochimica et Biophysica Acta (BBA)—Molecular Cell Research*.

[22] Okouchi M., Okayama N., Aw T. Y. (2005). Hyperglycemia potentiates carbonyl stress-induced apoptosis in naïve PC-12 cells: relationship to cellular redox and activator protease factor-1 expression. *Current Neurovascular Research*.

[23] Wyss M. T., Jolivet R., Buck A., Magistretti P. J., Weber B. (2011). In vivo evidence for lactate as a neuronal energy source. *The Journal of Neuroscience*.

[24] Avogaro A., Nosadini R., Doria A. (1990). Substrate availability other than glucose in the brain during euglycemia and insulin-induced hypoglycemia in dogs. *Metabolism*.

[25] Zheng H., Zheng Y., Wang D. (2016). Analysis of neuron-astrocyte metabolic cooperation in the brain of db/db mice with cognitive decline using 13C NMR spectroscopy. *Journal of Cerebral Blood Flow & Metabolism*.

[26] Strumia E., Pelliccia F., D'Ambrosio G. (2012). Creatine phosphate: pharmacological and clinical perspectives. *Advances in Therapy*.

[27] Mccormack S. E., Shaham O., Mccarthy M. A. (2013). Circulating branched-chain amino acid concentrations are associated with obesity and future insulin resistance in children and adolescents. *Pediatric Obesity*.

[28] Waagepetersen H. S., Sonnewald U., Larsson O. M., Schousboe A. (2000). A possible role of alanine for ammonia transfer between astrocytes and glutamatergic neurons. *Journal of Neurochemistry*.

[29] Niciu M. J., Kelmendi B., Sanacora G. (2012). Overview of glutamatergic neurotransmission in the nervous system. *Pharmacology Biochemistry and Behavior*.

[30] Bak L. K., Schousboe A., Waagepetersen H. S. (2006). The glutamate/GABA-glutamine cycle: aspects of transport, neurotransmitter homeostasis and ammonia transfer. *Journal of Neurochemistry*.

[31] Zhang H., Gu X. F. (2005). A study of gene expression profiles of cultured embryonic rat neurons induced by phenylalanine. *Metabolic Brain Disease*.

[32] Zhang Y., Gu X., Yuan X. (2007). Phenylalanine activates the mitochondria-mediated apoptosis through the RhoA/Rho-associated kinase pathway in cortical neurons. *European Journal of Neuroscience*.

[33] Hohmann S. (2015). An integrated view on a eukaryotic osmoregulation system. *Current Genetics*.

[34] Oenarto J., Görg B., Moos M., Bidmon H.-J., Häussinger D. (2014). Expression of organic osmolyte transporters in cultured rat astrocytes and rat and human cerebral cortex. *Archives of Biochemistry and Biophysics*.

[35] van der Graaf M., Janssen S. W. J., van Asten J. J. A. (2004). Metabolic profile of the hippocampus of Zucker Diabetic Fatty rats assessed by in vivo 1H magnetic resonance spectroscopy. *NMR in Biomedicine*.

[36] Kantarci K. (2007). 1H magnetic resonance spectroscopy in dementia. *British Journal of Radiology*.

[37] Horská A., Farage L., Bibat G. (2009). Brain metabolism in Rett syndrome: age, clinical, and genotype correlations. *Annals of Neurology*.

[38] Michel V., Yuan Z., Ramsubir S., Bakovic M. (2006). Choline transport for phospholipid synthesis. *Experimental Biology and Medicine*.

[39] Williams K. T., Schalinske K. L. (2007). New insights into the regulation of methyl group and homocysteine metabolism. *The Journal of Nutrition*.

[40] Wainfan E., Poirier L. A. (1992). Methyl groups in carcinogenesis: effects on DNA methylation and gene expression. *Cancer Research*.

[41] Surtees R., Hyland K., Smith I. (1990). Central-nervous-system methyl-group metabolism in children with neurological complications of HIV infection. *The Lancet*.

[42] Washburn S. E., Caudil M. A., Malysheva O. (2015). Formate metabolism in fetal and neonatal sheep. *American Journal of Physiology-Endocrinology and Metabolism*.

