# Supplementary material for: High Glucose-Induced PC12 Cell Death by Increasing Glutamate Production and Decreasing Methyl Group Metabolism
Source: Biomed Res Int. 2016 Jun 19;2016:4125731. doi: 10.1155/2016/4125731 (PMC4930799; doi:10.1155/2016/4125731)
Supplement: Supplementary file 1 — In the Supplementary Material, typical 1H NMR spectra in extracellular extracts of the control and high glucose (HG) groups were illustrated in Figure S1. A clear separation was observed between the control and HG groups by multivariate analysis (PLS-DA) based on the extracellular metabolite profiles and the contributed metabolites were identified from its loading plot (Figure S2). In addition, Table S1 lists the changes in extracellular metabolite levels between the control and HG groups. [file 4125731.f1.doc]

High glucose-induced PC12 cell death by increasing glutamate production and decreasing methyl group metabolism

Minjiang Chen^1,2^, Hong Zheng^1^, Tingting Wei^1^, Dan Wang^1^, Huanhuan Xia^1^, Liangcai Zhao^1^, Jiansong Ji^2^, Hongchang Gao^1,*^

^1^ School of Pharmaceutical Sciences, Wenzhou Medical University, Wenzhou 325035, China

^2^ Lishui Central Hospital, the Fifth Affiliated Hospital, Wenzhou Medical University, Lishui 323000, China

Minjiang Chen and Hong Zheng contributed equally to this work.

*Corresponding author. E-mail: gaohc27@wmu.edu.cn (H.C. Gao).

**Table S1.** Comparison of changes in extracellular metabolites levels between the control and HG groups.

| No. | Metabolites | |r|b | Variation^a^ | |
| --- | --- | --- | --- | --- |
|  |  |  | CON^c^ | HG^d^ |
| 1 | Isolecucine | 0.88 | -8.22±0.11 | -9.57±0.08^***^ |
| 2 | Valine | 0.50 | -9.07±0.60 | -6.96±0.19^*^ |
| 3 | Ethanol | 0.80 | -32.02±1.96 | -20.92±3.39^*^ |
| 4 | Fucose | 0.51 | 0.73±0.09 | 0.38±0.17 |
| 5 | Lactate | 0.62 | 25.84±0.51 | 31.54±0.36^***^ |
| 6 | Alanine | 0.89 | 7.35±0.15 | 2.57±0.13^***^ |
| 7 | Acetate | 0.99 | 0.44±0.05 | -0.12±0.19^*^ |
| 8 | Pyroglutamate | 0.97 | -3.82±0.16 | -3.92±0.15 |
| 9 | Methionine | 0.87 | -2.01±0.04 | -2.31±0.05^***^ |
| 10 | Lysine | 0.93 | -7.30±0.21 | -9.34±0.28^***^ |
| 11 | Creatine phosphate | 0.89 | -0.63±0.02 | -0.35±0.07^**^ |
| 12 | Creatine | 0.71 | -0.42±0.02 | -0.13±0.04^***^ |
| 13 | Choline | 0.97 | -1.81±0.05 | -1.18±0.07^***^ |
| 14 | Myo-Inositol | 0.92 | -1.66±0.10 | -2.26±0.16^*^ |
| 15 | Threonine | 0.98 | -1.24±0.08 | -2.03±0.05^***^ |
| 16 | Tyrosine | 0.90 | -2.65±0.03 | -2.77±0.03^*^ |
| 17 | Phenylalanine | 0.86 | -1.44±0.03 | -1.90±0.03^***^ |
| 18 | Pyridoxine | 0.12 | -1.05±0.76 | -0.24±1.36 |
| 19 | Tryptophan | 0.79 | -0.27±0.03 | -0.41±0.05^*^ |
| 20 | Histidine | 0.46 | 0.04±0.01 | 0.04±0.01 |
| 21 | Formate | 0.45 | 0.23±0.00 | 0.24±0.01 |

^a^ the change of metabolite level in the extracellular medium relative to the blank medium; ^b^ the absolute value of correlation coefficient obtained from PLS-DA; ^c^ control group (25 mM glucose); ^d^ high glucose group (75 mM glucose). Significant level: ^*^p<0.05, ^**^p<0.01, ^***^p<0.001.


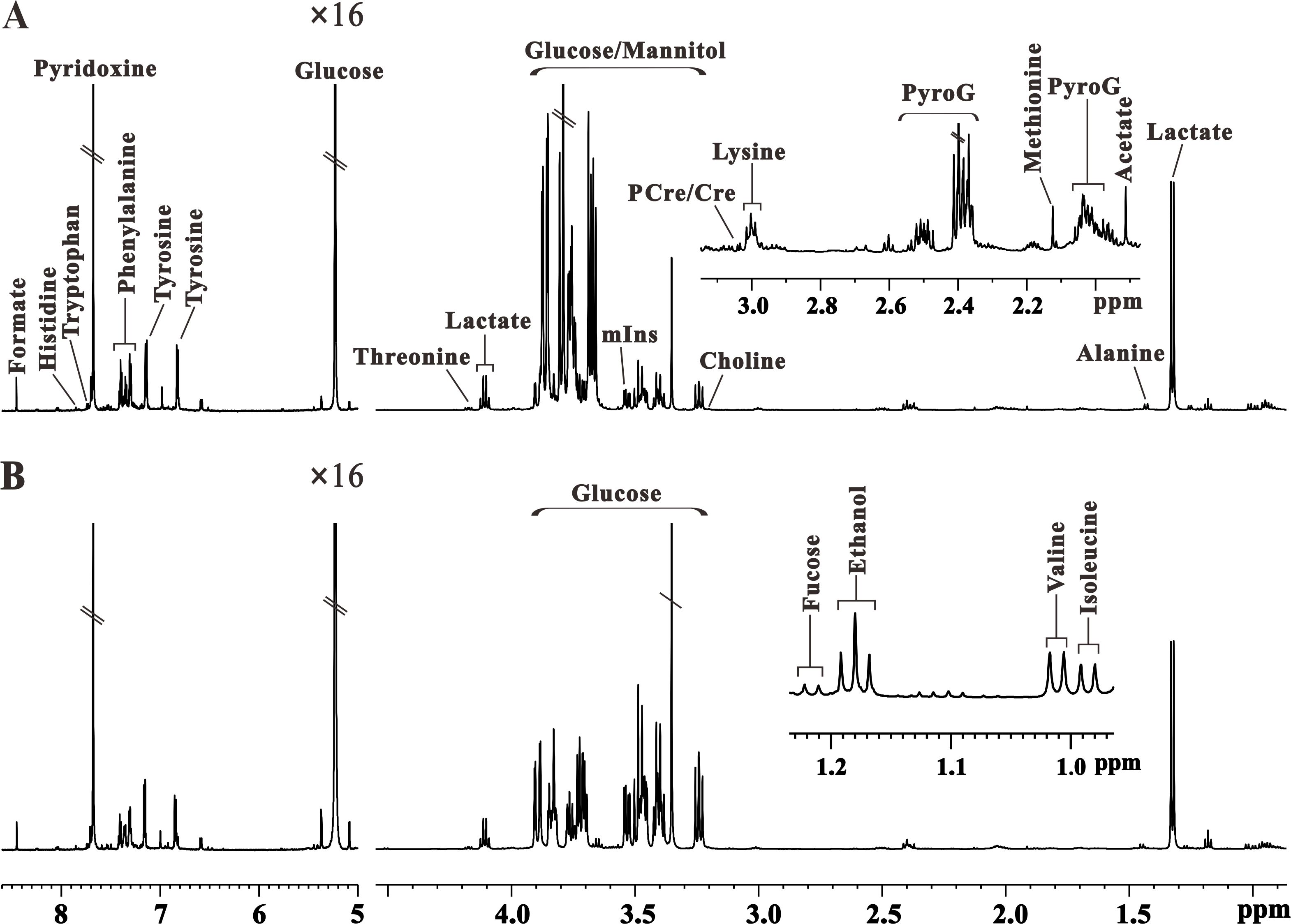


**Fig. S1.** Representative 600 MHz 1D ^1^H NMR spectra obtained from extracellular extracts in the control (A) and HG (B) groups. The abbreviation of metabolites: PyroG: pyroglutamate; PCre: creatine phosphate; Cre: creatine; mIns: myo-inositol.


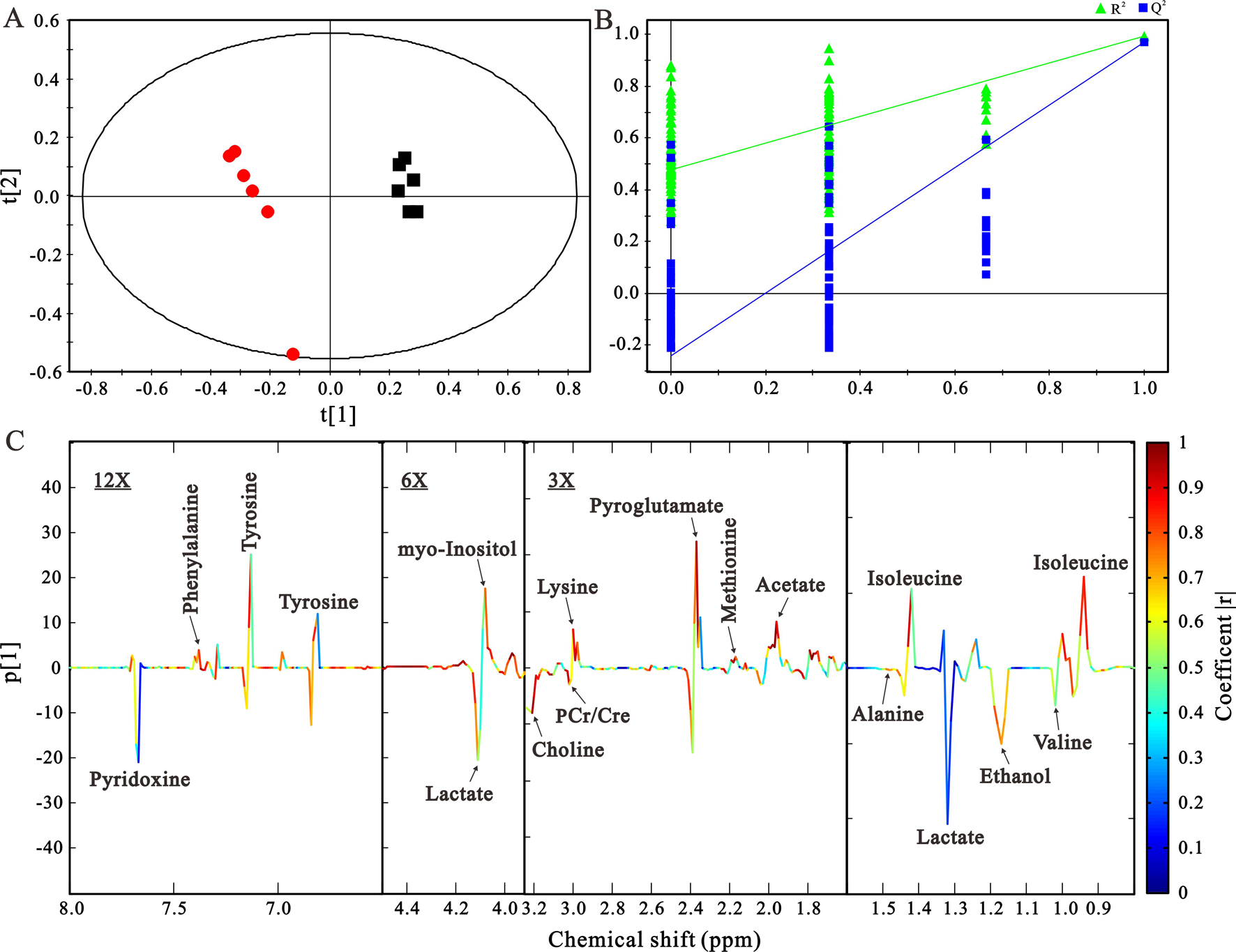


**Fig. S2.** PLS-DA results obtained from NMR-based extracellular metabolome in the control (■) and HG (●) groups: (A) score plot; (B) validation plot by permutation tests (200 cycles); (C) loading plot colored according to the absolute value of the correlation coefficient.
